# Grade 3 Giant Cell Tumour of the Distal Humeral Epiphysis Treated with Intralesional Curettage, High Speed Burring and Bone Grafting: A Case Report

**DOI:** 10.5704/MOJ.2211.020

**Published:** 2022-11

**Authors:** RW Nyffeler, K Ziebarth

**Affiliations:** 1 Department of Shoulder and Elbow Surgery, Orthopädie Sonnenhof, Bern, Switzerland; 2 Division of Pediatric Trauma and Orthopedics, University Hospital of Bern, Bern, Switzerland

**Keywords:** giant cell tumour, elbow, high speed burring, curettage, bone grafting

## Abstract

Giant cell tumours of bone are benign but locally aggressive neoplasms involving the epi- and metaphysis of long bones. Tumours of the distal humeral epiphysis with cortical disruption and invasion into the joint and the soft tissues are rare and generally treated with wide resection and joint reconstruction. We present the case of a 19-year-old woman in whom such a tumour was successfully treated with intralesional curettage, high speed burring and autologous bone grafting. The patient underwent regular clinical and radiological follow-up. Ten years after the procedure she had no signs of tumour recurrence or degenerative changes, and she was pain free and had a normal elbow function.

## Introduction

Giant cell tumours of bone are benign but locally aggressive tumours that nearly always arise within the epiphysis. They may rapidly increase in size and spread into the metaphysis or erode the cortex and expand into the joint. They are treated surgically, most often with curettage, either alone or in combination with additional mechanical procedures and/or chemical or thermal adjuvants^1^. Wide resection and reconstruction have been recommended for tumours that have extended through the cortex (Campanacci Grade 3 lesions) and are in close relationship to joint capsules. However, the functional long-term results of these reconstructions are not well known.

We therefore preferred a joint-preserving procedure for the treatment of a grade 3 lesion in a young and active woman. She consented to the publication of her medical history and pictures in a scientific journal.

## Case Reports

**Fig. 1.**
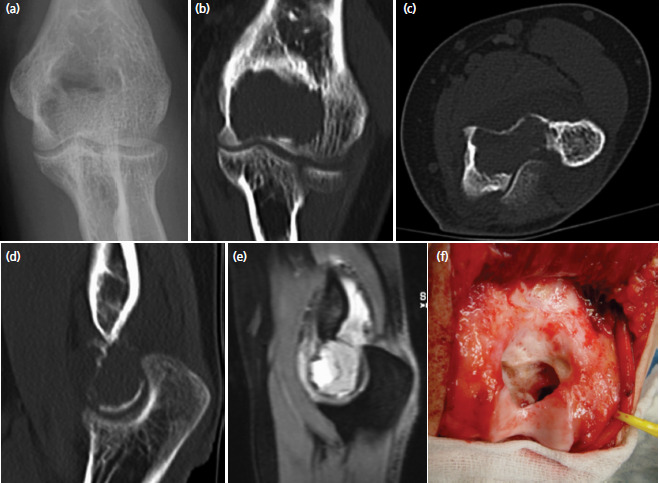
Pre- and intra-operative images revealing the big lytic lesion in the epiphysis of the left elbow: (a) anteroposterior radiograph, (b) coronal, (c) axial and (d) sagittal CT-images, (e) sagittal MRI, and (f) posterior view after intralesional curettage and high-speed burring. The subchondral bone of the trochlea and the medial epicondyle was severely thinned out and posteriorly disrupted. The ulnar nerve was marked with a vessel loop.

A 19-year-old, healthy women without previous medial problems consulted her general practitioner because of pain and decreased range of motion in her left elbow for six months. Standard radiographs showed a lytic lesion in the trochlea. She was referred to us for further assessment and treatment. The posteromedial aspect of the elbow was slightly swollen and sensitive to pressure. Skin temperature and colour were normal. Flexion was restricted to 100°, the extension deficit was 40°. Pronation and supination and peripheral sensibility and motor function were normal. Additional magnetic resonance images and computed tomography scans of the elbow revealed a locally destructive tumour in the distal humerus, involving almost the whole trochlea (Fig. 1). The cortical bone adjacent to the olecranon fossa was perforated and the tumour penetrated into the posterior compartment of the joint. It expanded proximally under the triceps but did not infiltrate this muscle. An open biopsy was performed, and the histologic analysis of tissue samples confirmed the diagnosis of a giant cell tumour. A CT thorax and scintigraphy of the skeleton showed no other lesions, especially lung metastases.

Due to the young age of the patient we decided to perform a joint preserving procedure. An olecranon osteotomy was made to expose the trochlea and the joint. The tumour was removed through the cortical defect in the posterior part of the trochlea with use of different curettes. A high-speed burr was then used to eliminate tumour remnants and reduce the risk of local recurrence. During the whole procedure the bone cavity was illuminated and examined with an arthroscope. Care was taken to not destroy the articular cartilage. The wound was regularly irrigated with saline solution and the instruments, as well as the drapes and gloves were changed. Thereafter the trochlear cavity was filled with autologous cancellous bone from the iliac crest. The bone was carefully compacted and the remaining cortical defect in the olecranon fossa was closed with two cortico-cancellous bone chips. The press fit was so good that no additional screws were necessary. Finally, the olecranon was reduced and stabilised with a tension band wiring. Post-operatively the elbow was protected in a removable splint for six weeks. During this time unloaded assisted motion exercises were allowed. Active motion and strengthening exercises were started after six weeks and the patient returned to work three months after the operation.

**Fig. 2.**
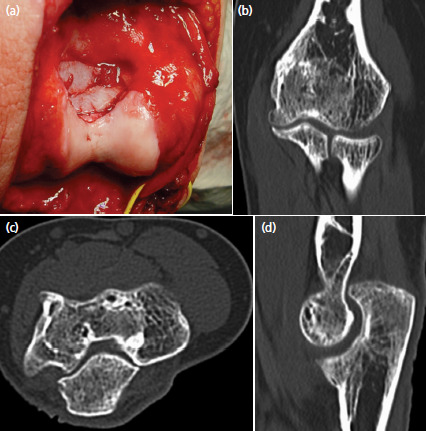
Intra- and post-operative images of the same elbow: (a) posterior view after autologous bone grafting. (b) Coronal, (c) axial and (d) sagittal CT-images made three years after tumour removal. The trochlea is anatomically shaped and filled with cancellous bone.

**Fig. 3.**
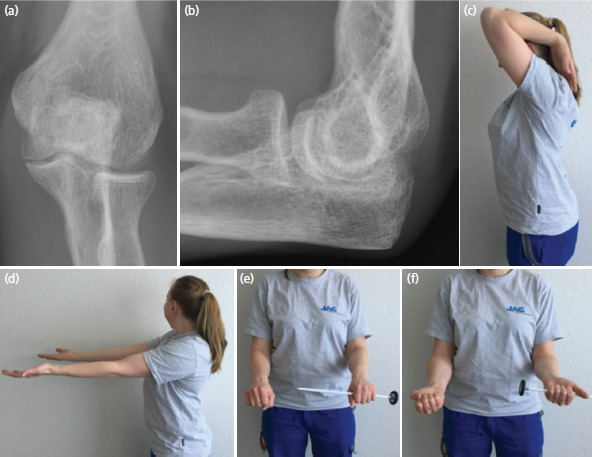
Radiographic and clinical result 10 years after tumour resection and autologous bone grafting. (a) Anteroposterior and (b) lateral radiographs of the elbow show a normal joint without tumour recurrence and without joint space narrowing. Photographs show normal (c) flexion, (d) extension, (e) pronation and (f) supination.

The tension band wiring slightly incommoded the slender patient and was therefore removed after eight months. At the same time, scar tissue in the olecranon fossa was debrided and the fossa was somewhat deepened with use of a spherical burr. This procedure allowed restoring full elbow extension. The histologic analysis of the scar tissue showed no tumour cells. Further follow-up examinations with clinical and radiographic investigations were performed after 6 months, 1, 2, 3, 5 and 10 years. An additional CT-scan was made after three years (Fig. 2). During the whole time and at the latest follow-up 10 years post-operatively the patient was doing well. She was pain free, had a normal range of motion, strength and worked full-time as an electrical installation technician. The Mayo Elbow Performance Score was 100 points. Standard anteroposterior and lateral radiographs of the elbow showed an anatomic joint with good incorporation of the bone graft, normal joint spaces and no signs of tumour recurrence or osteoarthritis (Fig. 3).

## Discussion

Several surgeons treated Grade 3 lesions in the distal humerus with en bloc resection alone^2^. Other surgeons resected the tumour and reconstructed the defect with bone cement, a structured autograft^3^, an allograft^4^ or an elbow prosthesis^5^. En bloc resection alone may compromise joint function. Bone cement has high mechanical strength and is therefore often used for large defects close to weight-bearing joints. In our case the subchondral bone was severely thinned out and the cartilage risked being damaged by the heat generation during curing of the bone cement. We therefore preferred meticulous mechanical cleaning of the whole cavity and filling the defect with autologous bone. Cui *et al*^3^ described six cases in which they used autogenous bone. In contrast to us, they did not use a high-speed burr to eliminate residual tumour cells adhering to the wall of the cavity but chemical adjuvants. These toxic substances, however, did not seem to have an adverse effect on the joint during the follow-up period, which ranged from 36 to 60 months.

We did not consider reconstructive measures with foreign materials in this young and active woman. Allografts may infect, fail to integrate, or resorb. Elbow prostheses are also susceptible to infections, and the polyethylene liner may wear out, resulting in prosthetic loosening. Elbow prostheses are therefore good for elderly patients with limited functional demands, but less suitable for young and active persons. The load limit recommended by most manufacturers of elbow prostheses is not compatible with physical work or normal sports activities. In young people, a joint-preserving treatment should therefore be sought. It has lower risks for complications, is less expensive and may provide better functional long-term results than wide resection and endoprosthetic reconstruction.
